# Broncho-Pneumonio

**Published:** 1896-08

**Authors:** N. A. Olive

**Affiliations:** Waco, Texas


### TEXAS MEDICAL JOURNAL,

ESTABLISHED JULY, 1885.

PUBLISHED MONTHLY.—SUBSCRIPTION $2.00 A YEAR.

VOL. XII. AUSTIN, AUGUST, 1896. NO. 2.

### Original Contributions.

For Texas Medical Journal.

### BRONCHO PNEUMONIA.

BY N. A. OLIVE, M. D., WACO, TEXAS.

Read before Waco Medical Association, April 14th, 1896.

SYNONYMS.—*Catarrhal Pneumonia*, *Lobular Pneumonia*.

DEFINITION: A catarrhal type of inflammation generally, at first, of scattered patches of lung tissue; or a pneumonic process of one or more lobules of one or both lungs.

The frequent association of this form of pneumonia with bronchial catarrh is said to explain the terms *broncho* and catarrhal applied to it. As the disease advances, or at a later period of the disease, the morbid process which, at first, is limited to scattered groups of air vessicles, attacks a wider area of lung tissue as a result of coalescence of the inflammatory nodules, producing more extended patches of consolidation.

The inflammatory products which fill the alveoli and produce the consolidation, are the epithelial cells of the alveoli themselves, and the bronchial tubes, and the exudation consequent on the pneumonic process.

CAUSE.—Age is prominent among the causes which superinduce the things antecedent to or leading up to broncho-pneumonia. Old age, with an enfeebled circulation, a damming up of the blood in the bronchial mucous membrane, with a sub-acute or chronic-congestive process, and a consequent bronchial catarrh, with shedding of the epithelial coating and exudation, more or less profuse, often lead to an aggravated form of the disease.

Infancy, with a circulatory apparatus, more imperfectly under the control of the central nervous system than at a later period of life, with its alternate period of excitement and depression, the arterioles now almost empty, and the next moment at flood tide; the tissues overflown; the nervous system unmindful of the peril, and if mindful, in a state akin to fright, and unable to respond to the demand made upon her, she is minus resources. This change of condition may not all take place in the short time I have taken to tell it, and the extreme opposites of the circulation may not occur in the short space of a moment. The language is simply employed to illustrate the conditions following opposite extremes that are so great a menace to the continuation of the vital processes during the period of infantile life.

Septic disease of the schneidarian or buccal mucous membranes of the pharynx, larynx or trachea, and the bronchial disorders, have been mentioned. All these are causes of the utmost importance. One of the most severe cases I ever saw recover was due to an aggravated case of aphthous stomatitis. Noxious gases may cause it, as also may smoke and other bronchial irritants act with other contributory causes and produce the disease. The accidental inhalation of foreign substance or particles of any kind may have a marked effect as an etiological factor. Extremes, with respect to climatic changes, are of no small import among the causative factors. Cold and damp weather is, perhaps, the most important.

The specific diseases, as measles, whooping cough, diphtheria, small-pox, and influenza, are frequently followed by broncho-pneumonia, and it is certainly the most important sequel of many of these cases. These, however, should have been mentioned as among the bronchial causes.

Physical condition is responsible to a certain extent. For instance, a depraved condition of the system, or a general lowering of all the vital forces, debility, from any cause, is important as a contributory cause of broncho-pneumonia. This, of course, weakens the muscles taking part in the respiratory act, thus favoring pulmonary collapse, which leads to this condition.

INVASION.—This disease, unlike lobar pneumonia, which is generally ushered in with a chill, a severe fit of vomiting, convulsion, or other unusual disturbance, is generally more or less insidious in its invasion, approaching with muffled step and stooping figure, endeavoring to hide its deadly mission. As before stated, the trachea, the larger bronchi, and the capillaries, may be successively affected in the approach of the disease to the pulmonary alveoli. It is, perhaps, doubtful if we ever have broncho-pneumonia without a capillary bronchitis ante-dating it. And some of the most able clinicians doubt the existence of a capillary bronchitis without its attendant broncho-pneumonia, and class them all under the head of broncho-pneumonia. The differential diagnosis, at least, is sometimes one of the most difficult things with which we come in contact. Especially is this the case in infancy.

SYMPTOMS.—The premonitory symptoms depend greatly upon the cause. When the disease is developing, or developed, the severity depends upon the extent of lung tissue involved, and nature of the disease to which it is due, and state of vitality of the subject attacked. Cough frequent and distressing, a rise of temperature, rapid respiration, more or less movement of the alæ nasi, dyspnœa, restlessness, violent effort of all the respiratory muscles, etc., are among the most common early symptoms of the disease.

During the course of a bronchitis of ever so mild a type, a rise of temperature, running up to 103–104, or 104½ or 105, is to be looked upon suspiciously, as it generally is present with the invasion of the lung tissue. It is the sign board marking the course of the disease. It is the beacon that bids us avoid breakers ahead. The rise of temperature is generally, however, gradual and goes on and up, reaching varying degrees of an abnormal height, depending upon various causes, the most prominent of which is amount of lung involved, the disease to which it is due, and rapidity of invasion. The fever runs an eccentric course, the hour of maximum being indefinite and may occur in the morning hours, or any time during the day.

The pulse, which is frequently at first full, strong, tumultuous, soon becomes quick, small and feeble. Respiration is shallow and the cough generally painful. The sputa is more or less bronchial in character; thick, tenacious, stringy mucuous, sometimes streaked with blood. In children under the fifth year there is seldom any sputa raised. The digestive apparatus often shares the general disturbance. There may be diarrhœa, and vomiting is occasionally a distressing symptom some time during the course of the disease. These things may supervene on looseness or indifference as to the character of food given.

The respiration, at first rapid and shallow, may develop from bad to worse. And the child may pass hours in a laborious struggle for its life. The picture is sad to the point of painfulness. The scene is distressing to the family and friends; and often, death in his mercy is welcome to close the scene, to mark a period of rest from the agony, the fearful torture of these long, anxious hours. When the darkness seems darkest, a faint ray of hope may yet steal in, which at first unrecognized, undetermined; only a short period of rest, a little while the patient seems better, but too soon lapses into its former condition. Now, another and somewhat longer period of rest and more marked relief. With this thing oft repeated, each period of rest longer and that of distress shorter and milder, the patient may finally be pronounced, usually, convalescent. He recovers, to the utter amazement of family and friends and surprise of the doctor. Before leaving the symptoms, we will recall the voice and cough. The voice, when the strength is sufficient to cry, is sometimes husky and the cough croupy, so much so, indeed, that you are unable to convince some knowing (?) ones that you are dealing with anything but a case of croup, either true or false.

### PROGNOSIS.

This depends on cause, amount of lung tissue involved, previous physical condition, age, former habits, any cachexia present, etc., etc. It is said that from infancy to the fifth year of life 20 per cent of those attacked die. In elder persons, those of broken constitutions and of vicious habits suffer most. They are not only more likely to contract the disease, but are more liable to weaken and give down at the perilous moment. With the class of subjects generally attacked, the prognosis is not the least encouraging.

### TREATMENT.

In treatment we are to bear in mind that the fever is not the disease by any means. It is simply an index to the severity of the pneumonic process. If need be treated the temperature is perhaps best controlled by the application of water suitable for the individual case. Cold water may be used fearlessly in conservative hands. There is no agent of more merit than this when properly administered. It ofttimes seems to arrest the progress of the disease, and it frequently obviates the necessity of giving opiates and other tranquilizing agents. And for my part I think there is seldom any indication for the coal tar derivatives in this class of disease. There is no doubt but they have killed many patients, and I have not seen evidence of any having been cured by them. There is not the least doubt, in my mind, that the profession has sinned greatly in the administration, the fearfully reckless administration, of these drugs during the past few years. No one would doubt their efficacy for good in some cases of disease, but I challenge any man to deny their equal potency for evil. Their analgesic effect, their exceedingly tranquilizing effect, render them the more popular, the more seductive, the more destructive.

This criminal negligence in the reckless administration of these drugs is censurable to an extreme degree and should be frowned down by the better class of our profession. The more culpable is he who pays obeisance to the secret formulary of whose proportions he knows nothing. Away with such an unscientific practice, such a rotten habit in the administration of drugs. And may the gods shower their wrath upon the college professor who signs a certificate attesting their merit. Now back to the treatment.

Husband the strength of the patient. Give good, nutritious diet; the mother's milk for a nursing babe can not be excelled. Alcoholic stimulants, too, are important. The malted milk, predigested milk, beef extract, etc., are among the various articles of diet that may be mentioned. Above all, give the patient plenty of fresh air, admit abundance of it; but maintain an equitable temperature at from 65° to to 70° F. Avoid draughts of air striking the patient, especially if the skin is moist and temperature low. Keep the room clear of chatter boxes. Gossip is not essential to the patient's recovery. Most of us like to have a chance for our lives. Some people are foolish enough to become offended when asked to retire from the sick room; but that is all right, if the patient's interests are involved, I do not think there is a member of this society who would hesitate to perform the delicate task of removing the oxygen-consumers When there is accumulation of mucus, etc., in the trachea, with an inability to throw it off and threatened suffocation, an emetic is used. Cardiac tonics or sedatives may be employed as demanded. Both are useful, but either can be terribly abused.

The ammoniacal preparations are generally useful as curative agents. Their use is too well understood to require comment.

Counter irritation, or blisters, I think, may be employed with benefit sometimes. However, their use is not to be generally recommended, and they should be dispensed with as much as possible.

This states the case briefly, and many important suggestions have been avoided, in deference to my hearers, as we hope not to be tedious, and some important points can be brought more forcibly out in the discussion.

